# Increased LDL susceptibility to oxidation accelerates future carotid artery atherosclerosis

**DOI:** 10.1186/1476-511X-11-4

**Published:** 2012-01-09

**Authors:** Toshinari Aoki, Tsueko Abe, Eiji Yamada, Takayuki Matsuto, Masahiko Okada

**Affiliations:** 1 Division of Clinical Preventive Medicine, Graduate School of Medical and Dental Sciences, Niigata University, 1 Asahi-machi, Chuou-ku, Niigata City 951-8510, Japan

**Keywords:** echo study, cohort, photometric absorbance, electrophoresis, HbA1c

## Abstract

**Background:**

We analyzed the causal relationship between LDL susceptibility to oxidation and the development of new carotid artery atherosclerosis over a period of 5 years. We previously described the determinants related to a risk of cardiovascular changes determined in a Japanese population participating in the Niigata Study, which is an ongoing epidemiological investigation of the prevention of cardiovascular diseases.

**Methods:**

We selected 394 individuals (169 males and 225 females) who underwent a second carotid artery ultrasonographic examination in 2001 - 2002 for the present study. The susceptibility of LDL to oxidation was determined as the photometric absorbance and electrophoretic mobility of samples that had been collected in 1996 - 1997. The measurements were compared with ultrasonographic findings obtained in 2001 - 2002.

**Results:**

The multivariate-adjusted model showed that age (odds ratio (OR), 1.034; 95% confidence interval (95%CI), 1.010 - 1.059), HbA1c (OR, 1.477; 95%CI, 0.980 - 2.225), and photometric O/N (OR, 2.012; 95%CI, 1.000 - 4.051) were significant variables that could independently predict the risk of new carotid artery atherosclerosis.

**Conclusion:**

The susceptibility of LDL to oxidation was a significant parameter that could predict new carotid artery atherosclerosis over a 5-year period, and higher susceptibility was associated with a higher incidence of new carotid artery atherosclerosis.

## Background

The oxidation of low-density lipoprotein (LDL) is widely believed to be an important event in the pathogenesis of atherosclerosis. Macrophages endocytose oxidized LDL in an uncontrolled manner, which results in the generation of cholesterol-laden foam cells that characterize atherosclerotic lesions [1,2]. The oxidation of LDL in vitro can be mediated by cells or mimicked in a cell free system using transition metal ions such as copper or iron as prooxidants [3,4]. Metal ions are usually prerequisite for LDL oxidation, at least in vitro. Catalytically active copper and iron are also present in human atherosclerotic lesions [5].

The initial step of LDL oxidation is the peroxidation of polyunsaturated fatty acids among LDL lipids. Fragments of modified fatty acids covalently attach to apolipoprotein B-100 (apoB-100) and neutralize its positively charged epsilon-amino acid group [2,6]. Another possible mechanism of the oxidation is that copper directly binds to apoB-100 at sites that are located at least on regions containing histidine [4]. Dietary antioxidants such as flavonoids and Vitamin E might help to inhibit copper binding to LDL [7,8].

One way of detecting oxidized LDL is to compare its photometric absorbance at 232 nm with that of native LDL [9]. An increase in photometric absorbance at 232 nm is due to the formation of conjugated dienes of fatty acid hydroperoxides. Continuous monitoring of the photometric absorbance of LDL under artificial modification shows a lag phase of which the rate is related to the antioxidant content and/or alterations in the surface structure [10-12]. This lag time is an independent parameter that can be used to evaluate the dynamics of LDL oxidation. Another popular method for detecting the process of oxidation is to measure the amount of thiobarbituric acid reactive substances (TBARS) that determine the malondialdehyde (MDA) content of oxidized LDL [13]. Electrophoretic mobility is also a conventional parameter that reflects an altered surface charge [14]. The susceptibility of LDL to oxidation is usually evaluated using the lag time, but this method is ad hoc and not standardized. Photometric absorbance and electrophoretic mobility are robust parameters that linearly correlate with the extent of LDL oxidation [15].

The role of LDL susceptibility in predicting new cardiovascular events is not yet fully elucidated. The present study analyzes the causal relationship between LDL susceptibility to oxidation and the development of new carotid artery atherosclerosis in a cohort that we have prospectively followed up for 5 years.

## Materials and methods

We previously described the determinants that are related to the risk of cardiovascular changes in a Japanese population that is participating in the ongoing epidemiological Niigata Study of the prevention of cardiovascular disease [16]. Full details of the study have been reported [17]. Briefly, 2164 individuals (1011 men and 1153 women; 23 - 89 years of age) were recruited from a single Japanese community in 1996 - 1997. The exclusion criteria at the initial entry comprised current and previous medication, history of diabetes mellitus, history of cardiovascular disease and fasting plasma glucose (FPG) > 0.69 mmol/L (125 mg/dL). All data were collected, analyzed and evaluated at a local health center. Plasma samples derived from venous blood anticoagulated with 3.4 mmol/L ethylenediamine tetra acetate (K_2_EDTA), were collected from all participants and immediately stored at -70°C.

The present study selected 394 individuals (169 males and 225 females) from the study cohort who underwent a second carotid artery ultrasonographic evaluation in 2001 - 2002 and met the criterion described below. The susceptibility of LDL to oxidation in the samples that had been stored since 1996 - 1997 was determined as photometric absorbance and electrophoretic mobility and the results were compared with the ultrasonographic findings obtained in 2001 - 2002. The Committee on Human Research, Niigata University School of Medicine, Japan approved the study protocol, and all participants provided written informed consent.

We prepared LDL (1.019 - 1.063 g/ml) using ultracentrifugation as follows. Sodium bromide (1.0 mL; 1.025 g/mL) containing 0.01% (w/w) NaN_3_, 0.3 mmol/L Na_2_EDTA, and 1 mmol/L benzamide hydrochloride was gently added to 2 mL of plasma, and the mixture was separated by ultracentrifugation at 400,000 × *g *(at the bottom of tube) at 4°C for 5 h using a Himac centrifuge CS100 (Hitachi Koki, Tokyo, Japan) and an S100AT5 rotor. The bottom fraction (2.0 mL) was then transferred to another tube and the density was increase to 1.063 using NaBr (1.151 g/mL). After repeated centrifugation at 400,000 × *g *at 4°C for 5 h, the top fraction was decanted and the container was filled with nitrogen and stored in the dark at 4°C until analysis. The LDL fractions were protected from the sun and room light, which can cause oxidation, and analyzed within 24 h of preparation.

Before oxidation, each LDL fraction was passed through a Sephadex G-25 column (PD-10™, Pharmacia Biotech, Uppsala, Sweden) to change the buffer to phosphate-buffered saline (PBS). The protein content of LDL samples was determined using a protein assay kit (Bio-Rad Laboratories, Inc., Tokyo, Japan) based on the Bradford method with bovine serum albumin as a standard, and adjusted to 100 μg/mL (final concentration). Copper sulfate (final concentration, 4 μmol/L) was added to each sample, incubated for 5 h, and then oxidation was stopped by separating the copper ions from LDL passage through a PD-10 column.

Trypsin (200 μg/ml) was added to native LDL (final protein concentration, 100 μg/ml) and incubated for 2 h at 37°C. Proteins that were non-specifically attached to LDL particles in portions of digested LDL were immediately resolved by 4 - 12% gradient polyacrylamide gel electrophoresis with sodium dodecyl sulfate (SDS-PAGE). The separated proteins were transferred to a polyvinylidene difluoride membrane (ATTO, Tokyo, Japan) and then bands were visualized by staining with Coomassie Brilliant Blue and sequenced at a separate facility (Takara Bio Inc., Otsu, Japan).

The susceptibility of LDL to oxidation was photometrically assessed using a DU 600 spectrophotometer (Beckman Instruments, Fullerton, CA, USA). Pairs of native and oxidized LDL samples were diluted with PBS to a protein concentration of 20 μg/mL and then absorbance was measured at 232 nm [14]. The ratio of the absorbance of oxidized LDL to that of native LDL (photometric O/N) was defined as a parameter of LDL susceptibility to oxidation.

We evaluated changes in surface charge as the electrophoretic mobility of native and oxidized LDL separated on 1.0% agarose gel films (TITAN GEL Lipoprotein, Helena Laboratories, Saitama, Japan) in 0.08 mol/L barbital buffer (pH 8.6), 90 V for 25 min according to the manufacturer's instructions. Lipoproteins were visualized by staining with 0.04 (w/v) Fat Red 7B in 95% methanol. The amount of protein in each applied sample was adjusted to a final concentration of 100 μg/mL. The relative mobility of LDL bands was automatically measured as distance in millimeters using an AE-6981GXCP ATTO COMBO II imager (ATTO, Tokyo, Japan). We defined the ratio of the distance migrated by oxidized to native LDL (electrophoretic O/N) as another parameter of LDL susceptibility to oxidation.

Carotid artery atherosclerosis was assessed as intima-medial thickness (IMT) measured by high-resolution B-mode ultrasonography according to Salonen et al. [18] using a TOSBEE scanner (Toshiba, Tokyo, Japan) equipped with a 7.5-MHz sector scanner probe (model PLF-705S; Toshiba) and a digital scan converter. The extracranial carotid arteries of supine participants were scanned from the lowest portion visible in the supraclavicular fossa to the carotid bifurcation. Determinations of IMT were based on the most severely affected site in either the right or left side of the neck. Affected sites included homogeneous thickening and localized plaque with or without calcification or ulceration or both. We did not classify the findings because homogeneous thickening and localized plaque can be difficult to discriminate. A trained sonographer performed all IMT measurements using image-morphometry software and an experienced physician validated all findings to eliminate concerns about inter-reader variability and erroneous reading.

In 1996 - 1997, blood collected after an overnight fast from an antecubital vein from seated subjects was tested within 3 h. The plasma insulin concentration was determined using an enzyme immunoassay (AxSYM Insulin, Dainabot, Tokyo, Japan) and FPG was measured using a glucose oxidase-oxygen electrode and Glucoreader-C (Shino-Test, Tokyo, Japan). Hemoglobin type A1c (HbA1c) was measured using high-performance liquid chromatography on an HLC-723GHb column (Tosoh, Tokyo, Japan). Serum total cholesterol (TC,) triglyceride (TG), and high density lipoprotein cholesterol (HDL-C) were enzymatically measured using a model 736 automatic analyzer (Hitachi, Tokyo, Japan). The amount of LDL cholesterol (LDL-C) was calculated from the amounts of TC, HDL-C, and TG using Friedewald's formula.

Seated systolic (SBP) and diastolic (DBP) blood pressure were measured at the upper arm using a sphygmomanometer. Subjects refrained from physical activity, eating and smoking for at least 30 min before measurement. We recorded SBP as the appearance of Korotkoff sounds, and DBP as their disappearance. Body mass index (BMI), calculated as weight (kg) divided by the square of height (m) [kg/m^2^] served as an index of overall adiposity.

The primary event was defined as IMT ≥ 0.8 mm in 2001- 2002 and IMT ≤ 0.7 mm in 1996 - 1997. Univariate analyses were performed using Student's *t *test and the Mann-Whitney test. The odds ratio (OR) of LDL susceptibility to oxidation to predict the risk of new carotid artery atherosclerosis was estimated by logistic regression modeling. Individuals with positive events in 2001 - 2002 were assigned 1 as the dependent variable in the logistic regression analysis; otherwise, 0 was assigned. To analyze the effect of potential confounders on the OR, we firstly adjusted the multivariate models for age only and then by including sex, LDL-C, HDL-C, TG, FPG, HbA1c, fasting insulin, SBP, DBP, BMI, photometric O/N, and electrophoretic O/N. A significant subset of independent risk factors was selected by backward stepwise elimination. All data were statistically analyzed using SPSS software, version 11.0J for Windows (SPSS Japan Inc). Values are expressed as means ± standard deviation (± SD). We calculated the 95% confidence interval (CI) for all ORs.

## Results

**Figure 1:**
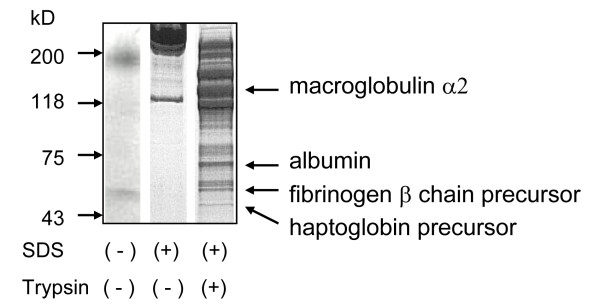
**SDS-PAGE of trypsinized LDL samples**. Macroglobulin α2, albumin, fibrinogen β chain precursor and haptoglobin precursor comprised four major bands identified by sequencing. Neither photometric nor electrophoretic O/N significantly differed between native and trypsinized LDLs.

Electrophoretic SDS-PAGE separation of trypsinized LDL revealed over 10 bands, among which, macroglobulin α2, albumin, fibrinogen β chain precursor and haptoglobin precursor predominated (Figure. 1). Since the electrophoretic profile was identical in ten randomly selected samples, we concluded that these proteins non-specifically attach to apoB-100. The photometric and electrophoretic O/Ns for both native and trypsinized LDL did not significanty differ.

Individual samples were repeatedly measured by both photometry (n = 20) and electrophoresis (n = 24). The intra-assay imprecision (coefficient of variation; CV) was 5.7% and 1.35%, respectively, and electrophoresis was more reproducible than photometry.

We used photometry and electrophoresis to determine the effects of temperature in two portions of the same sample stored at -70°C and +4°C for 14 days. Differences between values for each pair of aliquots were within the limits of intra-assay imprecision.

Table 1 shows the 1996 - 1997 characteristics of the participants with and without new carotid artery atherosclerosis that developed during the 5-year follow-up. Participants with carotid artery atherosclerosis were older and had higher levels of FPG and HbA1c. No other variables were significantly associated.

**Table 1:** Baseline characteristics of 394 participants in 1996 - 1997

Characteristic	IMT in 2001-2002		*P*
		
	≤ 0.7 mm	≥ 0.8 mm	
n	241	153	
Age	54.8	57.9	0.001
Sex (male/female) (n)	98/143	71/82	0.262
LDL cholesterol (mmol/L)	2.92 (0.76)^a^	3.07 (0.73)	0.068
HDL cholesterol (mmol/L)	1.46 (0.37)	1.46 (0.44)	0.975
Triglyceride (mmol/L)	1.11 (0.71)	1.20 (0.69)	0.223
Fasting plasma glucose (mmol/L)	5.27 (0.66)	5.44 (0.92)	0.035
HbA1c (%)	5.01 (0.6)	5.16 (0.6)	0.012
Fasting insulin (pmol/L)	29.4 (17)	31.1 (32)	0.492
Systolic blood pressure (mmHg)	125 (19)	126 (19)	0.401
Diastolic blood pressure (mmHg)	77 (10)	77 (9)	0.512
Body mass index (kg/m^2^)	22.6 (2.7)	22.9 (2.7)	0.298
Photometric O/N	1.90 (0.31)	1.95 (0.29)	0.156
Electrophoretic O/N	2.38 (0.29)	2.37 (0.25)	0.597

^a^Mean (standard deviation).

Table 2 provides two logistic regression models for predicting the risk of carotid artery atherosclerosis incidents with 13 variables including both O/Ns. There was no significant variable in the age-adjusted model and also in the multivariate-adjusted model except age. Analysis by backward stepwise elimination, in which the criterion for determining variables to be removed from the model was the likelihood ratio, showed that age (OR, 1.034; 95%CI, 1.010 - 1.059); HbA1c (OR, 1.477; 95%CI, 0.980 - 2.225); and photometric O/N (OR, 2.012; 95%CI, 1.000 - 4.051) were significant variables that could independently predict the risk of carotid artery atherosclerosis (Table 3).

**Table 2:** Multivariable odds ratios of risk factors at baseline for future increase in IMT

Characteristic^a^	Age-adjusted model^b^		Multivariate model^c^	
	
	OR (95% CI)^d^	*P*	OR (95% CI)	*P*
Age			1.035 (1.009 - 1.062)	0.009
Sex (male/female) (n)	0.744 (0.491 - 1.129)	0.165	0.732 (0.454 - 1.179)	0.199
LDL cholesterol (mmol/L)	1.005 (0.998 - 1.012)	0.147	1.006 (0.998 - 1.014)	0.144
HDL cholesterol (mmol/L)	1.000 (0.987 - 1.013)	0.998	1.010 (0.994 - 1.026)	0.234
Triglyceride (mmol/L)	1.002 (0.998 - 1.005)	0.371	1.001 (0.997 - 1.005)	0.670
Fasting plasma glucose (mmol/L)	1.012 (0.996 - 1.027)	0.137	1.001 (0.983 - 1.021)	0.877
HbA1c (%)	1.440 (0.960 - 2.160)	0.078	1.421 (0.878 - 2.298)	0.153
Fasting insulin (pmol/L)	1.017 (0.996 - 1.070)	0.531	1.013 (0.958 - 1.071)	0.646
Systolic blood pressure (mmHg)	1.000 (0.989 - 1.011)	0.985	0.995 (0.979 - 1.012)	0.589
Diastolic blood pressure (mmHg)	1.006 (0.985 - 1.027)	0.596	1.009 (0.978 - 1.041)	0.574
Body mass index (kg/m^2^)	1.035 (0.959 - 1.119)	0.376	1.026 (0.937 - 1.123)	0.584
Photometric O/N	1.919 (0.959 - 3.838)	0.065	1.943 (0.931 - 4.056)	0.077
Electrophoretic O/N	1.031 (0.479 - 2.220)	0.937	0.908 (0.386 - 2.135)	0.825

^a^Data at 1996-1997. ^b^Data adjusted for age. ^c^Variables in model were age, sex, LDL-cholesterol, HDL-cholesterol, triglyceride, fasting plasma glucose, HbA1c, fasting insulin, systolic blood pressure, diastolic blood pressure, BMI, photometric O/N, and electrophoretic O/N. ^d^OR, odds ratio; CI, 95% confidence interval.

**Table 3:** Odds ratios of variables selected by backward stepwise elimination

Characteristic^a^	Multivariate OR (95% CI)^b,c^	*P*
Age	1.034 (1.010 - 1.059)	0.005
HbA1c (%)	1.477 (0.980 - 2.225)	0.062
Photometric O/N	2.012 (1.000 - 4.051)	0.050

^a^Data in 1996-1997; ^b^OR, odds ratio; CI, confidence interval; ^c^Variables evaluated for elimination were age, sex, LDL-cholesterol, HDL-cholesterol, triglyceride, fasting plasma glucose, HbA1c, fasting insulin, systolic blood pressure, diastolic blood pressure, BMI, and photometric O/N, and electrophoretic O/N.

**Figure 2:**
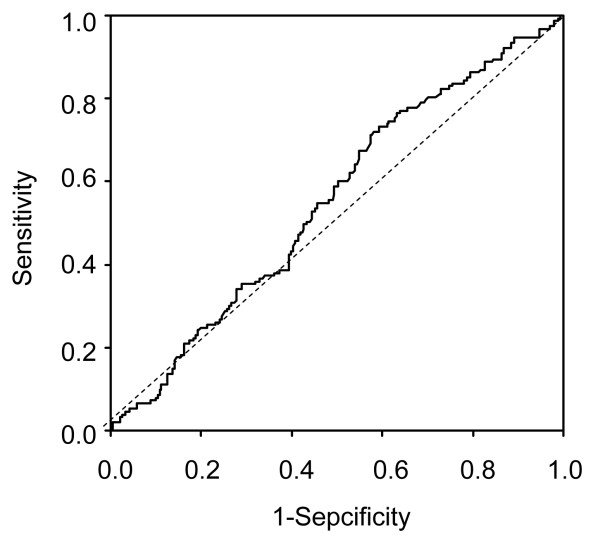
**ROC curves of photometric O/N in all participants for predicting future carotid artery atherosclerosis**. AUC value was 0.552 (P = 0.084). Sensitivity and specificity were 0.719 and 0.548, respectively at photometric O/N cutoff of 1.8.

Figure 2 shows two receiver operating characteristic curves (ROC) representing the sensitivity and specificity of photometric O/N. The area under the curve (AUC) were 0.552 (P = 0.084). At the cutoff points of photometric O/N = 1.8, 1.9 and 2.0, the sensitivity and specificity were 0.719 and 0.415, 0.536 and 0.548 and 0.373 and 0.647, respectively.

## Discussion

The present study found that LDL susceptibility to oxidation in combination with age and HbA1c was a significant parameter that predicted new carotid artery atherosclerosis over a period of 5 years. Higher susceptibility was associated with a higher incidence of carotid artery atherosclerosis.

The oxidative process initiated after copper ions are added to LDL particles is extremely complex [10,14,19]. Lipid peroxidation starts within 30 min, peaks at 2 h, and then the amount of products decreases. Modifications are accelerated after 60 min, but apoB-100 is quite difficult to modify. Phospholipids initially undergo a structural change and then conjugated diene breakdown products accelerate modification. ApoB-100 thus modified or its fragments accelerate further phospholipid modification. Although the direct binding of copper ions to apolipoproteins before these oxidative processes begin is accepted as fact, it cannot be observed by existing methods. Because conjugated diene formation empirically peaks within 2 - 5 h [14,20,21], we executed the measurements after a 5-h incubation. The photometric values of frozen and fresh LDL samples did not significantly differ, indicating that the integrity of the LDL samples used in the present study was preserved during storage.

The differing susceptibility of LDL to oxidation among individuals can be explained as follows. One is that LDL consists of a continuum of particles that can be classified according to their physical and chemical properties such as particle size, buoyant density, chemical composition, surface electrical charge and hydrodynamic behaviour [22]. Many studies have demonstrated that small dense LDL particles are easily oxidized and closely correlate with future atherosclerotic events. For example, Holvoet et al. reported that adding a parameter such as circulating oxidized LDL to the established risk factors might improve cardiovascular risk prediction [23]. Lie et al. reported that circulating oxidized LDL significantly correlates with LDL particle size in families with familial combined hyperlipidemia [24]. However, a study of diabetic patients found that plasma levels of oxidized LDL correlate only with the duration of diabetes independently of LDL size [25]. Kiechl et al. demonstrated that plasma levels of oxidized phospholipids on Lp(a) particles measured at baseline in an unselected population derived from the general community predicted the development of cardiovascular events over a 10-year prospective follow-up period [26]. Plasma LDL susceptibility to oxidation depends not only on the structural features of LDL but also on lifestyle characteristics such as daily consumption of fruits, vegetables, supplements or drugs [27-30]. The mechanism and atherogenesis of LDL oxidation are still not completely understood, and the theory of oxidized LDL remains controversial.

A crucial issue could be copper binding sites that become easily saturated not only by copper but also by antioxidants. Epidemiological studies suggest that a high intake of dietary antioxidants such as vitamin C, vitamin E, β-carotene is associated with a reduced risk of cardiovascular disease [7,8]. The non-radical decomposition of hydroperoxide (LOOH) can also modify the progress of peroxidation [31,32]. This indicates that individuals have some LDL that has already undergone an oxidative modification, which might increase the susceptibility of LDL to oxidation. Lankin et al. described the interesting findings that therapy with a 3-hydroxy-3-methylglutaryl-coenzyme A-reductase inhibitor (simvastatin) is associated with the intense accumulation of peroxidation products on the LDL of patients with coronary heart disease, despite having a decreased cholesterol level [33].

The present study has the following limitations. We defined cardiovascular events based on ultrasonographic measurements of the carotid artery and we did not include definitive variables such as angiographic findings. The participants in the Niigata study were initially healthy men and women without a history of cardiovascular diseases. Carotid artery ultrasonography is widely recognized as a valid non-invasive surrogate for detecting atherosclerosis, and several variables have already been directly implicated in increasing IMT [34]. Given this information, ultrasonography was the logical choice for the present study. The samples that we analyzed were frozen once and stored at -70°C for years. Although the experimental values did not significantly differ between fresh samples and those stored for 2 weeks at -70°C, the influence of temperature should be further clarified in the future.

In conclusion, we found that high susceptibility of LDL to oxidation was a significant parameter for predicting the likelihood of future carotid artery atherosclerosis.

## Authors' contributions

T Aoki, EY and TM carried out the preparation of LDL and the measurements, and performed the statistical analysis. T Abe participated in the ultrasonography of carotid artery. MO conceived of the study, participated in its design and coordination, and helped to draft the manuscript. All authors read and approved the manuscript.

## Competing interests

The authors stated that there are no conflicts of interest regarding the publication of this article. This study was funded by Denka Seiken Co. Ltd. Research funding played no role in the study design; in the collection, analysis, and interpretation of data; in the writing of the report; or in the decision to submit the report for publication.
